# Investigating the Correlation Between Corrosion-Induced Bolt Head Damage and Preload Loss Using Ultrasonic Testing

**DOI:** 10.3390/s25144491

**Published:** 2025-07-19

**Authors:** Jay Shah, Hao Wang, Abhijit Mukherjee

**Affiliations:** 1 Centre of Advanced Infrastructure and Transportation, Rutgers-The State University of New Jersey, Piscataway, NJ 08854, USA; 2 Department of Civil and Environmental Engineering, Rutgers-The State University of New Jersey, Piscataway, NJ 08854, USA; 3 School of Civil and Mechanical Engineering, Curtin University, Perth, WA 6102, Australia

**Keywords:** bolted joints, corrosion, ultrasonic testing, bolt head damage, interfacial contact, non-destructive testing

## Abstract

The integrity of bolted components primarily relies on the quality of interfacial contact, which is achieved by maintaining prescribed bolt torque levels. However, challenges arise from corrosion-induced bolt head damage, potentially compromising the bolt preload, and quantifying such effects remains unanswered. Many studies often compare bolt corrosion’s effects to bolt loosening as both affect the interfacial contact stresses to some extent. This technical study aimed to investigate whether a correlation exists between the impact of bolt head damage and the different levels of bolt torque. Guided wave ultrasonic testing (UT) was implemented for this investigation. Laboratory experiments were conducted to monitor the transmission of ultrasonic signals across the bolted interface first during the bolt-tightening process. Once the highest bolt torque was achieved, the process was repeated for a simplified corrosion scenario, simulated by artificially damaging the bolt head in a controlled manner. The analysis focused on studying the transmission of signal energy for both scenarios. The findings revealed different trends for the signal energy transmission during bolt tightening, which are subjective to the inspection frequency. On the contrary, even at an advanced level of bolt head damage corresponding to 16% mass loss, no clear or monotonic trend was observed in the total transmitted energy. While the total energy remained relatively stable across all inspection frequencies, distinct waveform changes, such as energy redistribution and the emergence of additional wave packets, were observed. The findings emphasize the need for more advanced waveform-based analysis techniques to detect and interpret subtle changes caused by bolt degradation.

## 1. Introduction

Bolted steel structures offer several advantages, including expedited installation and efficient recycling post-decommissioning. To ensure their long-term structural integrity, it is crucial to maintain the desired clamping force between the bolted components, which is achieved by applying a prescribed amount of bolt torque. The quality of the interfacial contact between these components directly influences the structure’s load-bearing capacity. However, steel joints are susceptible to rust formation in corrosive environments, leading to potential damage to the bolt head in the form of mass loss, which can compromise the clamping force. Quantifying the impact of such damage on structural integrity remains a challenging task, necessitating the continuous development of methodologies to assess joint conditions in corrosive environments effectively.

Several non-destructive inspections have explored the nature of interfacial contact in bolt-tightening and -loosening processes [1,2,3,4,5,6,7], but research on joint corrosion remains relatively limited [8,9,10]. Broadly, two main concepts have been extensively examined in these studies. Firstly, when bolt preload changes, the bolt undergoes geometric alterations, leading to axial and radial strains [11]. Moreover, corrosion-induced mass loss in the bolt also affects its strain [12]. Secondly, any variation in bolt preload directly influences the interfacial contact, which is often addressed in terms of the contact area developed due to the clamping force corresponding to the applied bolt torque [13]. Monitoring changes in bolt strain or contact area can be achieved through either direct methods, such as equipping the bolt with strain gauges [14] or smart sensors [15,16], or indirect approaches, like studying vibrations [17,18] in the joint to infer changes in the contact area and bolt strain. Each approach has advantages, and a fundamental understanding of their application is essential in selecting the most optimal method for investigating specific problems.

Wang et al. [11] conducted a study using radial strain gauges to explore the relationship between bolt preload and strain at the top surface of the bolt head. Their findings revealed a linear correlation between radial strain and bolt preload. In a similar investigation, Ahn et al. [12] employed strain gauge-fitted bolts to examine the loss of clamping force resulting from corrosion-induced bolt head damage, simulated by manual damage in a controlled manner. They established correlations between strain reduction and the volume of bolt head loss. Building upon a similar concept, Kong et al. [19] further expanded the research in an accelerated corrosion environment, where joints were corroded in a salt spray chamber. While the direct approach of using strain gauges has shown promising results, it may encounter limitations, especially in multi-bolt joints. Identifying and repairing of defective sensors in operational conditions can be challenging, potentially complicating real-world applications. Additionally, equipping bolts with sensors might necessitate damaging sections of the bolt, compromising the joint’s inherent load-bearing capacity.

The indirect approach of monitoring joint behavior through guide wave-based inspections is diverse and widely used. Among the standard techniques, vibration-specific features such as modal shapes [20] are employed for low-frequency joint inspections, while wave velocity [21], time of flight [22], amplitude [23], and phase changes [24] are utilized for high-frequency inspections. Modal shapes are known to be sensitive to changes in the global vibrational pattern of the joint but are generally less effective in detecting localized defects, particularly during their incipient stage, which is of great interest to the structural health monitoring community. In contrast, wave propagation in different components of the bolted joints has demonstrated higher sensitivity to changes in bolt preload.

In guided wave inspection, the ultrasonic signals are either actuated and received directly on the bolt or transmitted through the bolted components to study their interaction with the bolted interface. However, using the bolt as the wave propagation medium may need more versatility, especially in corroded joints, as transducer-based inspections rely on proper contact quality with the subject, which can be challenging with damaged bolt heads. Additionally, this method becomes time-inefficient in multi-bolt structures when monitoring individual bolts. To address these challenges, wave propagation across the bolted interface by actuating and receiving ultrasonic signals on the bolted components instead proves more relevant for studying bolt corrosion.

For bolted steel plate joints, the generation and reception of ultrasonic signals on the plates is generally well accepted. This allows the cumulative monitoring of wave interaction with the bolted interface, especially in the case of multiple bolts. Methodologies rely mainly on deciphering the interfacial contact-related changes in the ultrasonic signal features such as amplitude, energy, phase, propagation velocity, and time of flight. Shah et al. [8] hypothesized through their experimental observations that the variations in the transmitted signal energy result from rust formation at the bolted interfaces. Their results also hinted towards a potential relation between the initial bolt torque and load-bearing capacity loss of corroded joints. Cui et al. [25] reported similar results on steel bolt corrosion connecting two marble plates. Wang [9] utilized the linear and non-linear shape wavelets to analyze the experimental outcomes of UT in a multi-bolt corrosion setting. A relatively advanced approach includes monitoring the frequency spectrum of the signal as bolt loss may induce clapping-like behavior at the interface, resulting in the generation of higher-order or modulated harmonics, also termed contact acoustic nonlinearity [26]. However, the investigations are mostly limited to bolt torque loss studies. These findings highlight an evident gap in the UT-based investigation of bolt corrosion, and additional efforts are needed to develop novel insights to capture the effects of bolt corrosion.

## 2. Objectives and Scope

The primary objective of this study is to investigate the influence of bolt head damage on interfacial contact using guided wave UT. Laboratory experiments are first conducted to monitor the transmission trend of ultrasonic signal across the bolted interface at different bolt torques. Efforts are made to expand relevant insights over a wide range of excitation frequencies. This analysis is then repurposed to monitor the effect of controlled damage to the bolt head. This scenario is intended to replicate the impacts of corrosion-induced damage to the bolt head. It is anticipated that some level of relationship may exist between the results of the bolt-tightening stage and the bolt head damage stage. If established, the potential influence of bolt head loss on interfacial contact can enhance the understanding of real-life corrosion impact on the integrity of bolted joints.

## 3. Laboratory Experiments

### 3.1. Guided Wave Analysis

**Figure 1:**
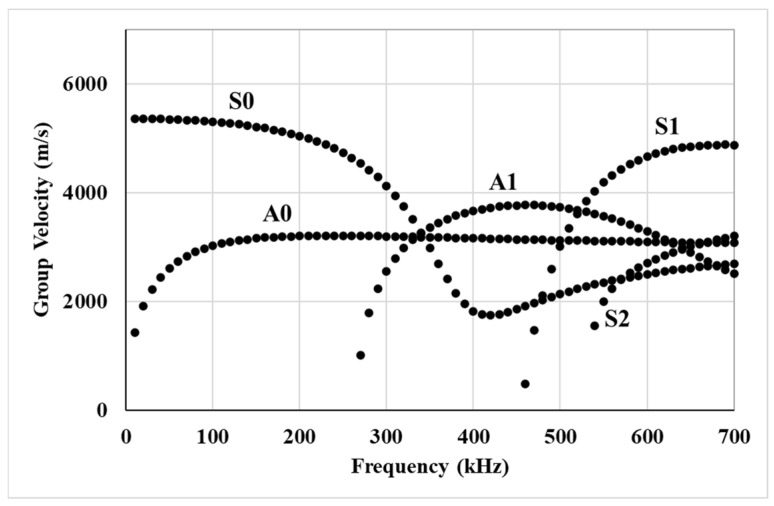
Group velocity profile of different guided wave modes in a 5 mm thick steel plate.

Typically, guided wave UT requires the identification and monitoring of specific guided wave modes in the transmitted signal. In thin plates, this requirement may restrict the range of inspection frequencies as multiple modes may co-exist together at higher frequencies. Figure 1 shows the group velocity profile for Lamb wave modes in the 5 mm thick plate used in this study. These curves were generated using GUIGUW 2.2 software with a steel properties database. It can be observed that higher-order symmetric and anti-symmetric modes start co-existing with zeroth-order modes beyond 280 kHz. Therefore, the interpretation of specific wave modes is challenging at higher frequencies. A potential meaningful contribution from higher-order modes is missed if restricted to a lower frequency range.

In this study, the inspection frequency range of 300 to 500 kHz was selected to investigate the behavior of higher-order guided wave modes and their interaction with local variations in the bolted interface. While lower frequency ranges (<280 kHz in this case) are commonly used due to their distinct group velocities, simpler mode structure and higher energy transmission, they have already been extensively studied in prior investigations [13], including those conducted by the authors [8,27,28]. In this study, the focus was shifted toward higher-frequency content, which enables the activation of shorter-wavelength modes that are more sensitive to small-scale damage such as early-stage bolt head deterioration. The upper frequency limit of 500 kHz was determined by the capabilities of the power amplifier used in the experimental setup. It could only amplify signals up to 500 kHz with a sufficient signal-to-noise ratio.

### 3.2. Experimental Setup

**Figure 2:**
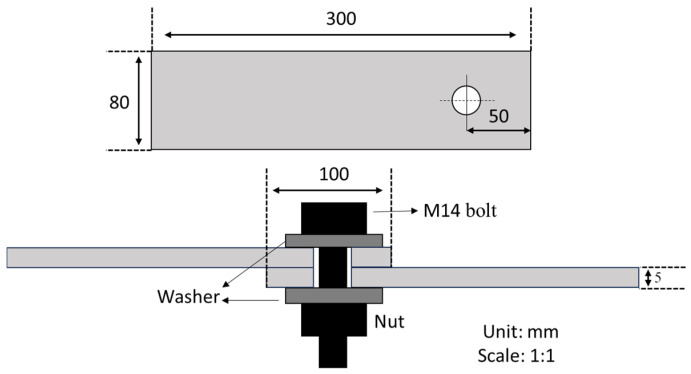
Schematic of the bolted specimen.

The bolted specimen comprises two 300 mm × 80 mm × 5 mm steel plates, which are bolted together using an M14 full-threaded steel bolt of grade 8.8, as illustrated in Figure 2. The joint has an overlapped length of 100 mm with a slight bolt clearance. To ensure uniform distribution of the bolt load during tightening, steel washers were utilized with both the bolt head and the nut.

**Figure 3:**
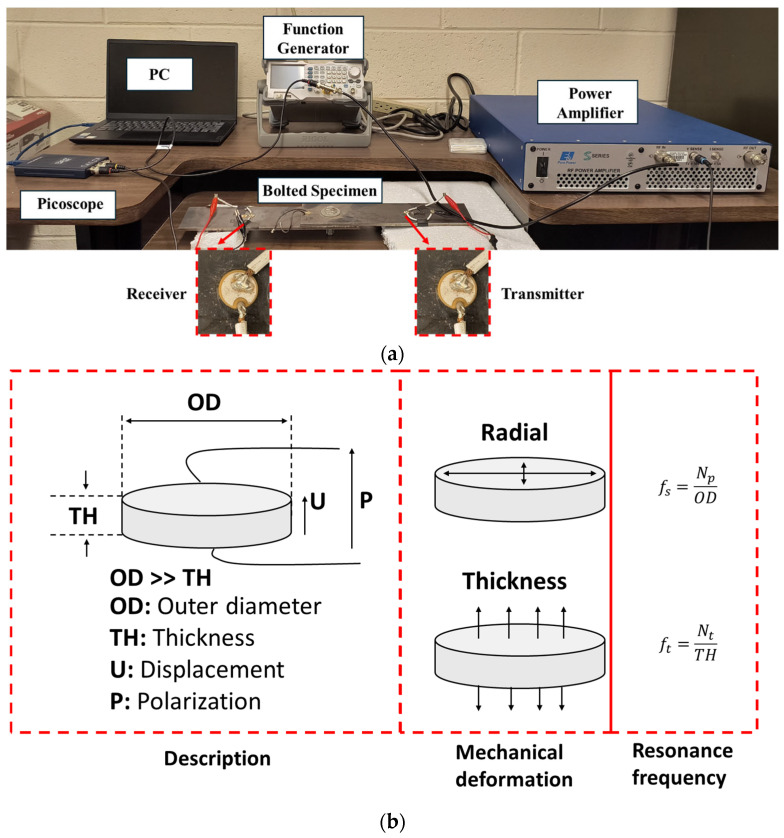
(**a**) Experimental setup for ultrasonic testing and (**b**) description of the piezoceramic disc.

For monitoring the effects on signal transmission due to bolt tightening and bolt head damage, a UT system, as illustrated in Figure 3a, was assembled to monitor the propagation of ultrasonic signals across the bolted interface. The signal generation unit consists of an arbitrary waveform generator (RIGOL DG1035Z, Portland, OR, USA), which was then amplified by a high-voltage power amplifier (E&I 1000S04, Electronics & Innovation, Ltd., Rochester, NY, USA). This amplified signal was transmitted through a piezoceramic disc (by PI Ceramics, Auburn, MA, USA) functioning as the transmitter, thus generating stress waves in the specimen. To record the wave propagation across the interface, an identical piezoceramic receiver was positioned on another plate. Figure 3b shows the schematic of the disc and its relevant description. The disc operates in d_33_ mode and the resonance frequencies along the radial and thickness directions can be calculated using relevant formulae included in the figure. The disc was made of ferroelectric soft material (PIC255) and its frequency coefficients for planar (f_p_) and thickness (f_t_) directions were 2000 Hz.m. For a 10 mm outer diameter and 1 mm thickness, these resonance frequencies correspond to 200 kHz in the radial direction and 2 MHz along the thickness direction. For additional details about the PIC255 material, ref. [29] can be referred to.

The cumulative distance between the transducers was set at 300 mm, providing sufficient wave–interface interaction before reaching the transducer. The received stress waves were then digitized using an oscilloscope (Picoscope 2204A, Pico Technology, Tyler, TX, USA) and recorded on a PC. The data acquisition software, called Picoscope, is readily available on the vendor’s website. It is to be noted that no signal amplification was used in the signal acquisition unit.

### 3.3. Monitoring the Effects of Bolt Torque

**Figure 4:**
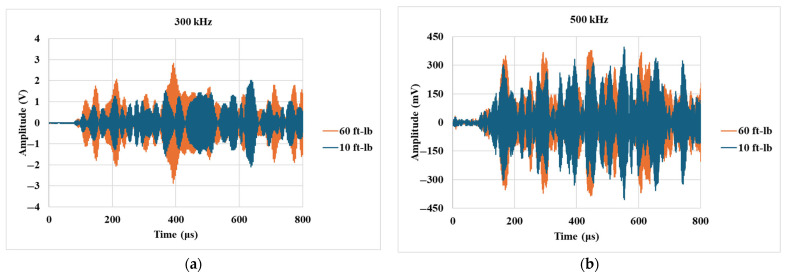
A comparison of signals recorded at the lowest and highest bolt torques for (**a**) 300 kHz and (**b**) 500 kHz signals.

The signal transmission across the bolted interface was recorded for the bolt-tightening stage, where the bolt torque was increased in steps of 5 ft-lb, starting from 10 ft-lb. The bolt was provided a maximum torque of 60 ft-lb using a handheld torque wrench, as progressing beyond this point could have potentially damaged the bolt thread. Different ultrasonic frequencies were chosen, ranging from 300 kHz to 500 kHz in steps of 50 kHz, to obtain elaborative insights on the effects of bolt torque on the interfacial contact. Figure 4 shows the A-scans for the transmitted signal at 300 kHz and 500 kHz. These signals are recorded up to 800 μs at a resolution frequency of 3.25 MHz, ensuring that they consist of the transmitted components around the central inspection frequency. Visual interpretation of Figure 4a reveals that the 300 kHz signal is reasonably sensitive to the change in bolt torque as the overall amplitude is relatively higher for 60 ft-lb of torque. In contrast, Figure 4b shows that the 500 kHz signal does not demonstrate much sensitivity to the change in bolt torque. A signal energy-based index was developed to quantify such changes.

**Figure 5:**
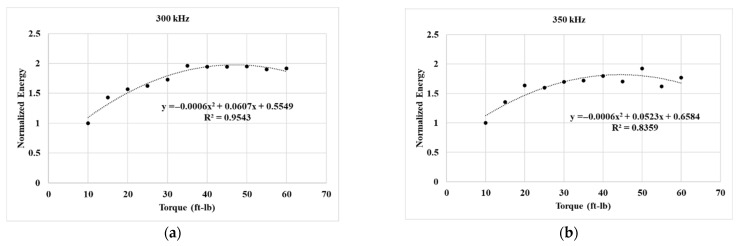

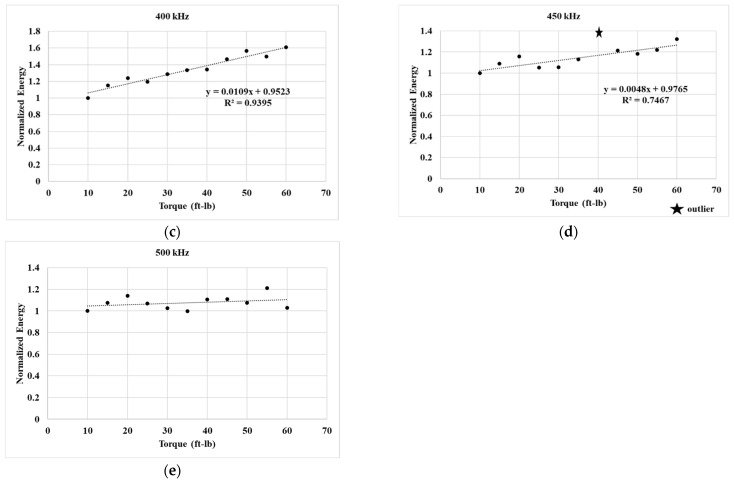
Experimental trend in transmitted signal energy at different bolt torques when inspected using (**a**) 300 kHz, (**b**) 350 kHz, (**c**) 400 kHz, (**d**) 450 kHz, and (**e**) 500 kHz.

A transmitted signal energy-based index was used to interpret the signals, as it remains the most interpretable metric despite increasing mode complexity at higher frequencies, where multiple modes begin to coexist. Transmitted energy is calculated by squaring the amplitude of the transmitted A-scans, as per Equation (1):

(1) $$Energy=\sum_{t_{i}}^{t_{f}}{\left(A\left(t\right)\right)}^{2}$$

where *A*(*t*) is the amplitude at the time *t*, and *t_i_* and *t_f_* are the lower and upper time bounds of the signal length. For this study, *t_i_* is 0 μs and *t_f_* is 800 μs. Figure 5 shows the experimental trend in transmitted energy at different bolt torques obtained using different inspection frequencies. All the transmitted energies are normalized relative to the energy at 10 ft-lb of torque.

For the 300 kHz inspection, an increase in the transmitted energy was observed from 10 ft-lb up to 35 ft-lb of torque. Beyond 35 kHz, the transmitted energy did not change and remained saturated close to twice the transmitted energy at 10 ft-lb of torque. The fitted curve with an R2 value of 0.95 confirms the quadratic nature of the relation between the bolt torque and the transmitted energy. This trend remained relatively identical for the 350 kHz inspection, where an R2 value of 0.83 was obtained. It is to be noted that these findings are relatively similar to the works of Wang et al. [13] and Li et al. [30], who correlated such changes with the increasing contact area between the plates. However, this energy-increasing trend changed drastically for 400 kHz and 450 kHz signals, where a clear linear relationship between the transmitted energy and bolt torque was observed. These insights have never been reported before, to the authors’ best knowledge. The 400 kHz inspection showed a strong linear correlation with an R2 value of 0.93, and 450 kHz showed a relatively weaker linear correlation, with an R2 value of 0.74. For the 500 kHz inspection, the overall energy transmission remained relatively unchanged, with an average normalized value of 1.1. Therefore, 500 kHz was not considered for further inspections in the study. Overall, the results demonstrate the benefits of using different inspection frequencies for bolt torque monitoring.

### 3.4. Discussion on Bolt Tightening and Energy Transmission

**Figure 6:**
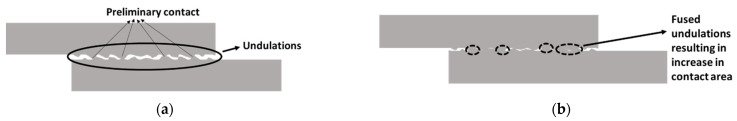
Microscopic interfacial contact upon application of torque with (**a**) lower transmission energy and (**b**) saturated transmission energy.

During the bolt-tightening process, the increase in the transmitted signal energy can be interpreted in the context of interfacial contact area development and interfacial contact stresses. For 300 kHz and 350 kHz, the increase in the transmitted energy at initial bolt torques may be linked to the development of a contact area between the plates. This contact area results from the increasing clamping force in the plates during the application of bolt torque. It has been consistently reported [13,31] that, at the microscopic level, the bolted interface is rough, as depicted by the undulations in Figure 6a. As bolt torque is applied, these undulations come into contact and fuse, developing the actual contact area as per Figure 6b. However, at higher bolt torques, the contact area saturates, resulting in the stagnation of transmitted 300 kHz and 350 kHz signals.

For the 400 kHz and 450 kHz cases, a nearly linear relationship was observed between the bolt torque and transmitted energy, even at higher torque levels beyond 35 ft-lb. In contrast, for the 300 kHz and 350 kHz cases, a saturation of transmitted signal energy was noted. This trend can likely be attributed to the influence of contact stress, which was examined in detail in the authors’ previous study [27]. In that study, signal transmission was analyzed in terms of both the development of the contact area and the stress within that area. It was found that while the contact area tends to remain relatively constant at high torque values, contact stress continues to increase across the entire torque range. This suggests that ultrasonic signals at 400 kHz and 450 kHz are more responsive to variations in contact stress compared to those at lower frequencies. Higher-frequency waves, due to their shorter wavelengths, are more sensitive to localized stress changes and fine-scale surface interactions at the interface. As a result, even in the absence of significant changes in contact area, increased stress may enhance mechanical coupling and reduce energy loss due to scattering or reflection, thereby improving energy transmission across the joint.

This distinction is important when interpreting ultrasonic responses, as lower-frequency signals are more likely to reflect changes in contact area, while higher-frequency signals are better suited to capturing subtle variations in contact stress.

It is to be noted that this study was carried out using a single specimen due to practical constraints, including the inability to achieve the same mass loss with handheld tools and extended lead times for acquiring specific transducer. While statistical validation through repeated trials was not feasible within the scope of this work, the experimental approach was based on prior investigations published by the authors. In an earlier study [28], no significant variation in transmitted signal energy was observed at 300 kHz during corrosion progression, suggesting a threshold behavior in energy response. These findings, observed under an electrochemical corrosion framework, are consistent with the trends observed in the current study. As such, the methodology used here is supported by existing evidence, and it is anticipated that similar trends would be observed in repeated trials under equivalent conditions. The relevant reference has been cited to support this consistency.

### 3.5. Determination of the Effects of Bolt Head Damage

**Figure 7:**
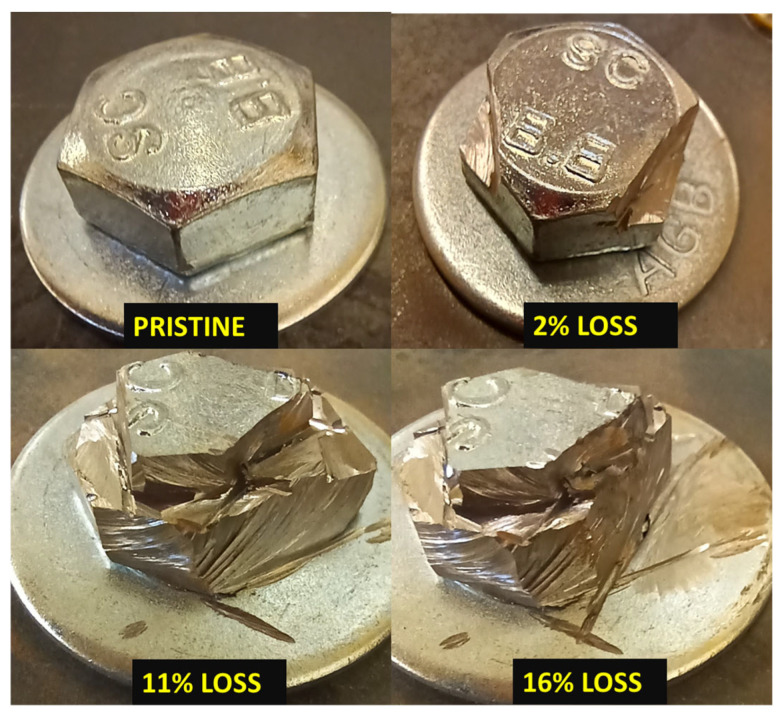
Bolt head condition at different mass loss.

Considering the observations from the bolt torque monitoring, it is anticipated that unique correlations between the bolt head damage and the transmitted energy may be observed. As outlined in this section, the bolt head was damaged in a controlled manner using a handheld cutting tool, as shown in Figure 7. Only the bolt head was damaged in the process, and the mass loss in the whole specimen was solely due to the material lost from the bolt head. The original mass of the pristine bolt, 56 g, was measured prior to the creation of the bolted joint. At each stage of bolt head damage, the specimen was weighed using a digital scale, and the mass loss was calculated by comparing it to the initial mass of the pristine specimen. After each quantifiable change in the specimen mass, UT was performed. The bolt head was damaged progressively until a mass loss of 16% was achieved. At this mass loss, the bolt head is severely damaged, and continuing further does not justify the use of UT as the bolt head can simply be inspected using a basic visual inspection. The initial state of the specimen had the highest torque of 60 ft-lb, which was achieved in the previous section.

The approach of using bolt head damage to represent corrosion was adopted from the work by Ahn et al. [12], who studied the impact of bolt head degradation on clamping force using tension test experiments. While it is acknowledged that actual corrosion involves complex electrochemical processes and multi-site deterioration, the objective here was to decouple these factors and investigate the direct influence of bolt head mass loss on guided wave transmission. This approach allows for a clearer interpretation of signal variations, which would otherwise be obscured in uncontrolled corrosion environments. Similar limitations of electrochemical corrosion setups were observed in prior studies by the authors [8,12], where the extent and location of damage could not be confined solely to the bolt head. Therefore, the adopted method was considered to offer a reproducible approach for establishing initial correlations between preload loss and ultrasonic signal behavior, which can serve as a basis for future modeling and validation under more realistic corrosion scenarios.

**Figure 8:**
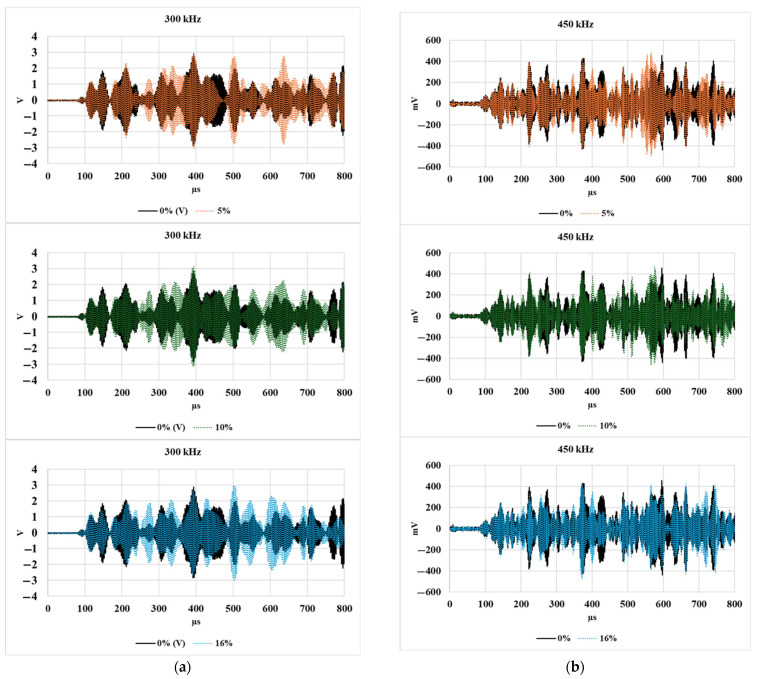
Comparison of recorded signals at 0%, 5%, 10%, and 16% levels of bolt damage for (**a**) 300 kHz and (**b**) 450 kHz signals.

Figure 8 shows the A-scan responses of transmitted signals for 300 kHz and 450 kHz inspection frequencies for varying levels of mass loss. Contrary to the typical expectation of a monotonic decrease in signal amplitude with increasing damage, a notable redistribution of signal energy was observed across different time windows. Rather than a uniform attenuation, distinct changes in the waveforms were seen, especially the emergence of new signal envelopes that were absent in the baseline (0% mass loss) case. At both frequencies, certain signal components appeared delayed or amplified as damage progressed. For example, in the 300 kHz panels, the signals corresponding to 10% and 16% mass loss show a clear shift and growth in later-arriving wave packets, indicating possible changes in wave scattering, mode conversion, or path alterations due to the evolving contact condition. Similar trends were observed for the 350 kHz and 400 kHz signals; however, they are not included here to avoid redundancy in the discussion.

These changes suggest that mass loss at the bolt interface does not simply weaken the transmission but alters the wave interaction dynamics, potentially exciting different guided wave modes or enabling energy to couple into paths that were previously less active. Such behavior highlights the complexity of interpreting A-scan data in bolted structures.

**Figure 9:**
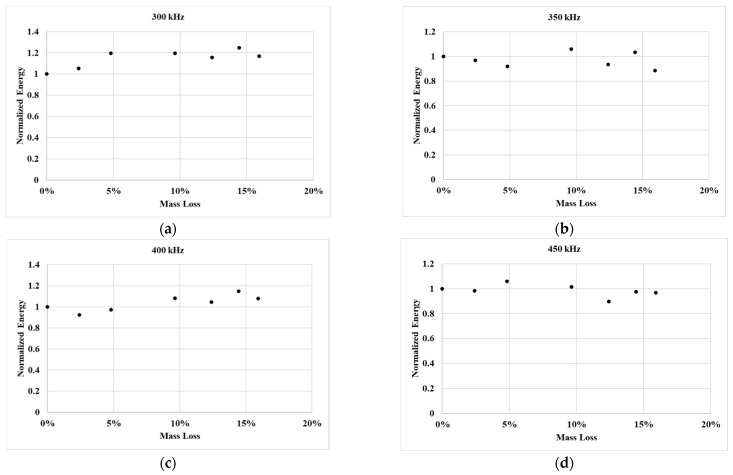
Experimental trend in transmitted signal energy at different bolt head damage states when inspected using (**a**) 300 kHz, (**b**) 350 kHz, (**c**) 400 kHz, and (**d**) 450 kHz.

Figure 9 presents the variation in normalized transmitted energy at different levels of mass loss, evaluated across multiple inspection frequencies. The energy was computed using Equation (1). Interestingly, despite increasing mass loss, the overall transmitted energy remains relatively consistent, showing no clear trend or monotonic decline. While minor fluctuations are observed, these are not sufficiently distinct to serve as reliable indicators of damage progression. This aligns with the waveform observations in Figure 8, where, rather than a simple loss of energy, a redistribution of energy across different time windows was observed. The presence of new wave packets and altered envelopes suggests that damage influences the mode content and temporal energy localization rather than causing a uniform energy reduction. Therefore, relying solely on transmitted energy as a damage metric may overlook more subtle, yet significant, waveform changes caused by evolving interface conditions.

### 3.6. Discussion on Bolt Head Damage and Energy Transmission

Understanding the effect of bolt head loss during the corrosion process, particularly under high torque, is of critical interest. Such understanding is essential for early detection of the loss of contact integrity resulting from corrosion-induced bolt head degradation. Surprisingly, despite high levels of mass loss, the total transmitted energy remained largely unchanged across all cases. However, as demonstrated in Figure 8, this does not imply the absence of changes in the signal. Instead, significant redistribution of wave energy and the emergence of new signal components were observed, pointing to complex underlying mechanisms that are not captured by energy-based metrics alone. This observation leads to several interesting hypotheses that can shed light on the underlying mechanisms:

- Residual stressed memory at the contact interface: The fusion of undulations at the plate interface during the bolt-tightening process may create localized regions that maintain mechanical coupling even when the bolt head undergoes substantial degradation. These fused areas could act as stable transmission paths, making the signal appear relatively unaffected despite physical damage.
- Limited role of the bolt head: The persistence of signal transmission could be attributed to redundancy in mechanical load paths. In bolted joints, especially under high preload, contact is not limited to the bolt head alone. Other components such as washers or the interface between the clamped plates may provide alternate transmission routes. These routes could remain intact even as the bolt head undergoes mass loss, thereby preserving the mechanical coupling necessary for ultrasonic transmission.
- Wave mode conversion: As bolt head damage progresses, the geometry and stiffness distribution at the interface change, which can affect how incident ultrasonic waves propagate through the structure. These changes may enable mode conversion. Additionally, irregularities at the degraded interface can introduce new scattering paths and reflections, resulting in time shifts and additional wave packets in the received signal. This aligns with the observations from Figure 9, where new signal patterns and delayed arrivals indicate that energy is being redirected rather than lost. Such effects can significantly alter the waveform shape while leaving the overall energy content relatively unchanged, making it crucial to evaluate waveform features beyond amplitude or energy.

In practical situations, corrosion may not be confined strictly to the bolt but may also affect surrounding materials, such as the plate surface near the bolt head. This can change the local stiffness and coupling characteristics in a way that redistributes the wave energy but does not necessarily attenuate it significantly. These coupled effects complicate the interpretation of energy-based metrics and suggest the need for localized signal analysis that accounts for subtle variations in wave behavior.

Exploring these hypotheses and conducting further experimental and numerical investigations will provide valuable insights into the observed behavior and contribute to a deeper understanding of the influence of bolt head damage on the ultrasonic inspection process.

## 4. Conclusions

This technical study aimed to investigate the potential correlation between bolt head damage and interfacial contact using UT. The transmission of signal energy was monitored across the interface during both the bolt-tightening process and the bolt damage process. The main findings of the study are as follows:

- During the bolt-tightening phase, the 300 kHz and 350 kHz signals showed a linear increase in the energy transmission at initial bolt torques; however, beyond 35 ft-lb, the energy transmission reached saturation and did not change for the higher torque values. In contrast, the 400 kHz and 450 kHz showed a linear correlation between the bolt torque and energy transmission at all the considered bolt torque values.
- The energy transmission recorded with a 500 kHz signal did not show any variation in the transmitted signal energy and stayed relatively unchanged for all the considered torque values.
- During the bolt head damage phase, the overall transmitted energy remained relatively unchanged, even at 16% mass loss. However, distinct changes were observed in the waveform, including the redistribution of energy across time windows and the appearance of additional wave packets that were not present in the undamaged condition, indicating altered wave paths and potential mode conversions at the interface.

Future scope of work

The findings indicate that different inspection frequencies interact distinctly with bolt torque, and that while energy transmission did show changes with increasing bolt head damage, the trends were not monotonic as initially expected. Instead of a consistent decrease in transmitted energy, complex waveform changes such as energy redistribution and the appearance of new signal features were observed. This highlights the need for more advanced analytical techniques capable of capturing and interpreting these subtle changes in signal characteristics across different stages of joint corrosion. Further investigations combining destructive testing with detailed numerical modeling could help establish clearer relationships between bolt torque, bolt damage, and ultrasonic response, ultimately improving the reliability of inspection methods for bolted joints.

## Data Availability

Dataset available on request from the authors.
